# Prothrombotic State in Asthma Is Related to Increased Levels of Inflammatory Cytokines, IL-6 and TNFα, in Peripheral Blood

**DOI:** 10.1007/s10753-017-0565-x

**Published:** 2017-04-20

**Authors:** Stanislawa Bazan-Socha, Lucyna Mastalerz, Agnieszka Cybulska, Lech Zareba, Romy Kremers, Michal Zabczyk, Grazyna Pulka, Teresa Iwaniec, Coenraad Hemker, Anetta Undas

**Affiliations:** 1 Department of Internal Medicine, Jagiellonian University Medical College, Krakow, Poland; 2 Department of Differential Equations and Statistics, Faculty of Mathematics and Natural Sciences, University of Rzeszow, Rzeszow, Poland; 3 Synapse Research Institute, Cardiovascular Research Institute Maastricht, Maastricht University, Maastricht, the Netherlands; 4 Centre for Medical Research and Technologies, The John Paul II Hospital, Krakow, Poland; 5 Allergy and Clinical Immunology Department, University Hospital, Krakow, Poland; 6 Institute of Cardiology, Jagiellonian University Medical College, 80 Pradnicka St., 31-202 Krakow, Poland

**Keywords:** Asthma, Thrombin generation, Fibrinolysis, IL-6, TNFα, Periostin

## Abstract

Recently, we have reported that asthma is associated with enhanced plasma thrombin formation and impaired fibrinolysis. The mechanisms underlying the prothrombotic state in this disease are unknown. *Our aim* was to investigate whether prothrombotic alterations in asthmatics are associated with inflammation. We studied 164 adult, white, stable asthmatics and 72 controls matched for age, sex, body mass index (BMI), and smoking. Plasma tumor necrosis factor α (TNFα), interleukin (IL)-6, and serum periostin were evaluated using ELISAs, and their associations with thrombin generation, fibrinolytic capacity, expressed as clot lysis time (CLT), and platelet markers were later analyzed. Asthma was characterized by 62% higher plasma IL-6 and 35% higher TNFα (both, *p* < 0.0001). Inflammatory cytokines were higher in sporadic and persistent asthmatics compared to controls, also after adjustment for potential confounders. IL-6 was inversely related to the forced expiratory volume in 1 s/vital capacity (FEV_1_/VC) spirometry index after correction for age, sex, and BMI. IL-6 and TNFα were associated with C-reactive protein in asthmatics (*β* = 0.6 [95% CI, 0.54–0.67] and *β* = 0.33 [95% CI, 0.25–0.41], respectively) and controls (*β* = 0.43 [95% CI, 0.29–0.57] and *β* = 0.33 [95% CI, 0.18–0.48], respectively). In asthma, IL-6 and TNFα positively correlated with the endogenous thrombin potential (*β* = 0.35 [95% CI, 0.28–0.42] and *β* = 0.15 [95% CI, 0.07–0.23], respectively) but not with CLT or platelet markers. However, TNFα predicted CLT in a multiple linear regression model. Periostin was not associated with any hemostatic parameters. Enhanced thrombin generation is driven in asthma by a systemic inflammatory state mediated by IL-6 and to a lesser extent TNFα, however, not periostin. TNFα might contribute to impaired fibrinolysis.

## INTRODUCTION

Inflammation and coagulation are biological processes that might interact with each other in physiological and pathological conditions [1]. Asthma is a heterogeneous disease, characterized by chronic airway inflammation, responsible for bronchial reversible obstruction and hyper-responsiveness [2]. There is growing evidence for heightened activation of blood coagulation in the airways of asthmatic subjects and procoagulant plasma protein leakage into bronchoalveolar space [3]. However, it is not known whether this local phenomenon might affect blood coagulation and/or clinical symptoms in asthma patients. A hemostatic imbalance in asthma has been postulated after a rising number of reports on the increased risk of thromboembolic events in asthmatics [4–7]. Recently, we have investigated parameters of blood coagulation, platelet activation, and fibrinolysis in a large cohort of asthmatics and demonstrated that this disease is characterized by enhanced plasma thrombin formation, associated with impaired clot lysis and blood platelet activation [8]. Prothrombotic state in asthmatics has also been documented by Sneeboer et al. [9], who showed increased thrombin generation, together with higher levels of plasminogen activator inhibitor-1 (PAI-1), D-dimer, von Willebrand factor, and plasmin-α_2_-antiplasmin complexes in this disease. In our study, the hypercoagulable state in asthma was potently and independently determined by increased blood C-reactive protein (CRP) concentrations [8]. Based on this finding, we hypothesized that a hypercoagulable state in asthma is related to the systemic inflammatory response largely mediated by interleukin (IL)-6, which is the most important inducer of the synthesis of acute phase proteins, including CRP [10]. Mononuclear phagocytes are the main source of IL-6; however, it is also produced by other inflammatory cells participating in asthma pathology, such as T and B lymphocytes, fibroblasts, and endothelial cells [10]. It has been shown that IL-6 might even be higher in the blood of asymptomatic asthmatics [11]. Moreover, clinical and laboratory data revealed that this cytokine is involved in the activation of the blood coagulation pathway. An intravenous bolus infusion of IL-6 in humans induced an increase in the plasma thrombin-antithrombin complexes and the prothrombin activation fragments F1+2, but it had no impact on fibrinolysis [12]. It has been also demonstrated that IL-6 levels in acute coronary syndrome are associated with prothrombotic plasma thrombin generation profile [13].

Tumor necrosis factor α (TNFα) is another cell signaling cytokine that rises in the acute phase reaction [10]. This cytokine interacts with endothelial cells permitting the egress on granulocytes into inflammatory loci and activation of these cells [10]. In healthy humans, the neutralization of endogenous TNFα attenuated a severe systemic inflammatory response, including cytokine release and activation of neutrophils [14]. In addition to mononuclear phagocytic cells, TNFα may be produced by neutrophils, activated lymphocytes, natural killer cells, endothelial cells, and mast cells; all of them are involved in the pathogenesis of asthma. TNFα was higher in severe asthmatics as compared to mild-moderate asthma subjects and controls [15]. This cytokine also has some prothrombotic properties. A single intravenous injection of recombinant TNFα into normal humans induced an early activation of factor (F)X, followed by a potent activation of prothrombin, probably *via* the release of tissue factor (TF) [16]. TNFα might also be involved in the regulation of fibrinolysis. Infusion of recombinant TNFα in human volunteers induced initial activation and subsequent inhibition of plasmin generation [17]. In patients with cancer, TNFα was capable of eliciting the release of a tissue-type plasminogen activator (tPA), urokinase-type plasminogen activator (uPA), and PAI-1 into circulation, indicative of the activation and rapid inactivation of fibrinolysis [18].

Periostin is an extracellular matrix protein that is generated, *e.g.*, by airway epithelial cells in response to IL-13. Serum periostin has been shown as an emerging biomarker of T helper type 2 (Th2)-driven inflammation, which is strongly associated with airway eosinophilia in severe asthma [19, 20]. To our knowledge, there have been no published studies analyzing the potential impact of periostin on blood coagulation; however, Th2 cytokines (*i.e.*, IL-4, IL-10, and IL-13) were shown to inhibit this process [21].

In the present study, we tested the hypothesis that an enhanced inflammatory state detectable in peripheral blood is associated with prothrombotic alterations observed in the circulating blood of asthmatics. Moreover, we speculated that Th2-driven inflammation, reflected by periostin concentrations, is involved in the prothrombotic state in this disease. Therefore, we investigated the associations between the previously analyzed parameters of a prothrombotic state in asthma [8] and concentrations of IL-6 and TNFα and periostin in peripheral venous blood.

## MATERIALS AND METHODS

### Patients and Controls

We studied 164 white adult patients with clinically stable asthma and 72 control subjects matched for sex, age, body mass index (BMI), and smoking habits. Inclusion and exclusion criteria were described previously [8]. Briefly, diagnosis of asthma was established based on a history of recurrent respiratory symptoms, including wheezing, shortness of breath, chest tightness, and cough, together with the documented post-bronchodilator increase in forced expiratory volume in 1 s (FEV_1_) of at least 12% and 200 ml from the baseline. Atopic status was confirmed by a positive skin prick testing for at least one inhaled allergen (Allergopharma, Reinbeck, Germany). Allergic asthma was diagnosed if the relevance of allergen exposure and its relation to symptoms were confirmed by the patient’s history [22]. All asthma medications, with the exception of omalizumab, were permitted. Asthma patients could not be exacerbated during the preceding 6 months. Severity of asthma was categorized according to the Global Initiative for Asthma (GINA) guidelines [22]. Spirometry was performed using a Jaeger, Master Screen spirometer on all enrolled subjects according to the American Thoracic Society standards. If basal spirometry showed bronchial obstruction, the bronchial reversibility test with 400 μg of albuterol was performed and parameters of the second spirometry were analyzed for further investigation.

Control subjects matched for age, sex, BMI, and smoking by frequency were enrolled from hospital personnel and relatives; all had normal spirometry and no history of allergic diseases or bronchial obstruction in the past.

The study was approved by the Ethics Committee of the Jagiellonian University.

#### Informed Consent

Informed consent was obtained from all individual participants included in the study.

### Laboratory Investigations

Fasting blood samples were drawn from the antecubital vein using minimal stasis between 8:00 and 11:00 A.M. Lipid profile, glucose, creatinine with estimated glomerular filtration rate, alanine aminotransferase, blood cell, and platelet count were assayed by routine laboratory techniques. Fibrinogen, high-sensitivity (hs) CRP, and immunoglobulin E (IgE) were measured as previously described [8]. Blood samples were drawn into tubes containing 0.109 mol/l sodium citrate (vol/vol, 9:1) and serum separation tubes, centrifuged 2000×*g* for 20 min at room temperature, within 2 h from sampling. The supernatant was frozen in aliquots and stored at −70 °C until analysis.

Commercially available immunoenzymatic assays were used to measure IL-6 and TNFα (R&D Systems, Minneapolis, MN, USA), as well as periostin (Phoenix Pharmaceuticals, Burlingame, CA, USA).

Methodology to determine plasma plasminogen, α_2_-antiplasmin, prothrombin, antithrombin, PAI-1, platelet factor 4 (PF4), and P-selectin, as well as determination of α_2_-macroglobulin, and clot lysis time (CLT), a global test of plasma fibrinolytic potential, was described previously [8].

To assess thrombin generation, we used the calibrated automated thrombogram (CAT) with computational model of thrombin dynamics and analyzed, *i.e.*, the maximum concentration of thrombin formed during the recording time (described as the “thrombin peak”) and the area under the curve which represents the “endogenous thrombin potential” (ETP), as described [8, 23].

### Statistical Analysis

The assumption of normality was verified with the Shapiro-Wilk test. All continuous variables were non-normally distributed and presented as median with upper and lower quartile. On figures, they were shown as median with interquartile range and maximum and minimum values. Continuous variables were compared by the Mann-Whitney *U* test, Kruskal-Wallis, and multiple repetition tests, as appropriate, while categorical variables by *χ* ^2^ test. To test the relationship between continuous variables, the univariate linear regression model was used. Because IgE had a skewed distribution, a 10 log-transformed was entered for a better fit of the model. Other variables were calculated in the original scale. The strength of the association of IL-6 and TNFα with ETP was analyzed by the test comparing the correlation coefficients. Independent determinants of CLT and ETP were established in a multiple linear regression model, built by a forward stepwise selection procedure, and verified by F Snedecor’s statistics, with *F* > 1. The *R* ^2^ was used as a measure of the variance. Log-linear analysis and unconditional multivariate logistic regression were performed to make adjustments for categorical variables (age [cut-off value of 50 years], sex, BMI [<25 and ≥25 kg/m^2^], hypertension, diabetes mellitus, coronary heart disease, smoking, and medications used, *i.e.*, statins, aspirin, antihypertensives, corticosteroids, montelukast, theophylline, and oral estrogens). To calculate odds ratios (ORs) with 95% confidence intervals (CIs), TNFα and IL-6 were divided using the value of 75th percentile in controls as a cut-off point.

*P* values <0.05 were considered statistically significant. Analysis was performed with STATISTICA 10.0 software package (StatSoft, Inc., Tulsa, OK, USA).

## RESULTS

### Patient Characteristics

Demographic and clinical characteristics of the subjects studied were given in detail in our previous publication [8]. Briefly, asthmatics and controls were well matched for age (53.5 [38.5–63] and 50.5 [39–58] years, *p* = 0.34), sex (male-to-female ratio 25.6 and 25%, *p* = 0.92), and BMI (26.6 [23.9–29.7] and 25.6 [23.2–29.1] kg/m^2^, *p* = 0.15), as well as other cardiovascular risk factors, including smoking habits and co-morbidities. Most asthmatics represented overweight, middle-aged women, with a predominance of allergic asthma (*n* = 94, 57.3%). The latter patients were younger, more often male with a lower prevalence of hypertension compared with non-allergic asthmatics. Twenty asthmatics (12.2%) were diagnosed with sporadic asthma, 36 (21.9%) with persistent mild, 45 (27.4%) with moderate, while 63 (38.4%) had severe asthma.

All persistent asthmatics (*n* = 144, 87.8%) received ICS, 124 (75.6%) long-acting β_2_-agonists, 40 (24.4%) montelukast, 26 (15.8%) theophylline, and 34 (20.7%) oral corticosteroids. All asthmatics on theophylline and all but one subject on montelukast received inhaled corticosteroids. Ten patients on montelukast (25%) and 13 on theophylline (50%) were also treated with oral corticosteroids.

### Laboratory Variables

Asthmatics had higher inflammatory markers, *i.e.*, hsCRP and fibrinogen, as well as α_2_-macroglobulin and PF4, compared with the control subjects (Table 1). Asthma was characterized by markedly increased ETP (1508.3 [1342–1680.7] *vs.* 1255 [1078.6–1454.2] nmol/l thrombin min, *p* < 0.0001), higher thrombin peak (283.6 [244.3–328.5] *vs.* 200.5 [154.1–229.6] nmol/l, *p* < 0.0001), faster rate of thrombin formation (time to peak) (5 [4.67–6] *vs.* 5.92 [5.17–7.13] min, *p* < 0.0001), together with higher total amount of prothrombin converted (904.7 [797–1026] *vs.* 848.1 [739.4–937] nmol/l, *p* = 0.03), and lower thrombin inactivation capacity (thrombin decay capacity) (0.63 [0.58–0.69] *vs.* 0.7 (0.6–0.75) min^−1^, *p* = 0.0004), as compared with control subjects. We observed impaired fibrinolysis in asthmatics, reflected by longer CLT (95.2 [71–117.4] *vs.* 83.2 [71.45–99] min, *p* = 0.001), and accompanied with higher plasminogen (106.7 [94.5–19] *vs.* 102.4 [96.5–107] %, *p* = 0.02) and PAI-1 (29.48 [21.74–42.54] *vs.* 24.3 [18.7–29.75] ng/ml, *p* < 0.0001), but unaltered antiplasmin (103 [96–114] *vs.* 100.3 [90.6–110.5] %, *p* = 0.09).

**Table 1:** A Summary of Selected Laboratory Variables in the Subjects Studied

	Asthma patients *n* = 164	Control subjects *n* = 72	*p* value asthma *vs.* controls	Allergic asthma *n* = 94	*p* value allergic asthma *vs.* controls	Non-allergic asthma *n* = 70	*p* value non-allergic asthma *vs.* controls	*p* value allergic *vs.* non-allergic asthma
hsCRP, mg/l	1.27 (0.59–2.55)	0.87 (0.29–2.9)	0.03	1.21 (0.56–2.3)	0.34	1.85 (0.62–2.78)	0.04	0.67
Fibrinogen, g/l	3.6 (3.0–3.95)	3.36 (2.73–3.57)	0.001	3.5 (3.0–3.9)	0.03	3.65 (3.1–4.1)	0.004	0.39
α_2_-macroglobulin, nmol/l	3.7 (3.23–4.22)	3.29 (2.89–3.57)	0.0002	3.78 (3.36–4.30)	<0.0001	3.54 (3.04–4.22)	0.02	0.22
Platelet factor 4, ng/ml	146.65 (129.87–156.91)	97 (87–112.5)	<0.0001	148.29 (129.61–157.85)	<0.0001	144.96 (130.13–156.49)	<0.0001	0.62
Immunoglobulin E, IU/ml	1.92 (1.37–2.37)	1.5 (1.39–1.84)	0.003	2.26 (1.91–2.61)	<0.0001	1.29 (1.24–1.68)	0.23	<0.0001
Eosinophils, /ul	166 (100–300)	ND	NA	180 (80–300)	NA	140 (100–250)	NA	0.7
Interleukin 6, pg/ml	4.55 (3.58–5.73)	3.06 (2.56–4.1)	<0.0001	4.43 (3.45–5.6)	<0.0001	4.83 (3.87–5.86)	<0.0001	0.14
Tumor necrosis factor α, pg/ml	3.93 (3–4.7)	2.91 (2.26–3.7)	<0.0001	4.02 (3.02–4.75)	<0.0001	3.8 (2.91–4.69)	<0.0001	0.69
Periostin, ng/ml	0.44 (0.32–0.6)	ND	NA	0.41 (0.32–0.56)	NA	0.45 (0.34–0.7)	NA	0.3

Data is presented as median with upper and lower quartiles

*ND* not done, *NA* not applicable, *hsCRP* high-sensitivity C-reactive protein, *n* number

### Inflammatory Cytokines

**Fig. 1:**
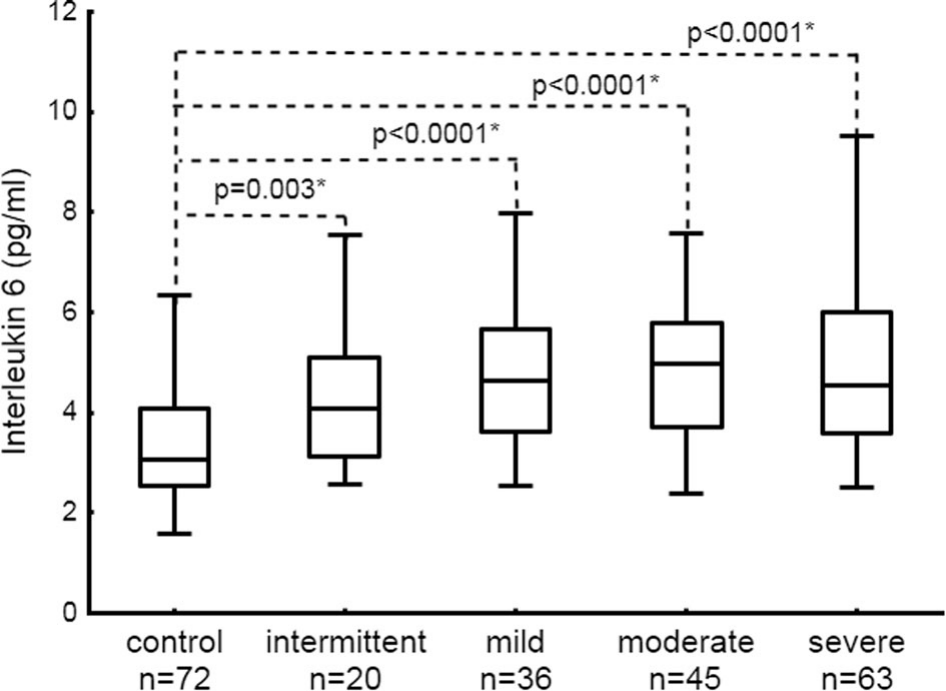
Distribution of interleukin 6 in controls and patients with intermittent, persistent mild, moderate, and severe asthma. Data is presented as median, interquartile range, and maximum and minimum values. Numbers on the graph represent *p* values in comparison to control. Statistically significant differences are marked with an *asterisk*.

**Fig. 2:**
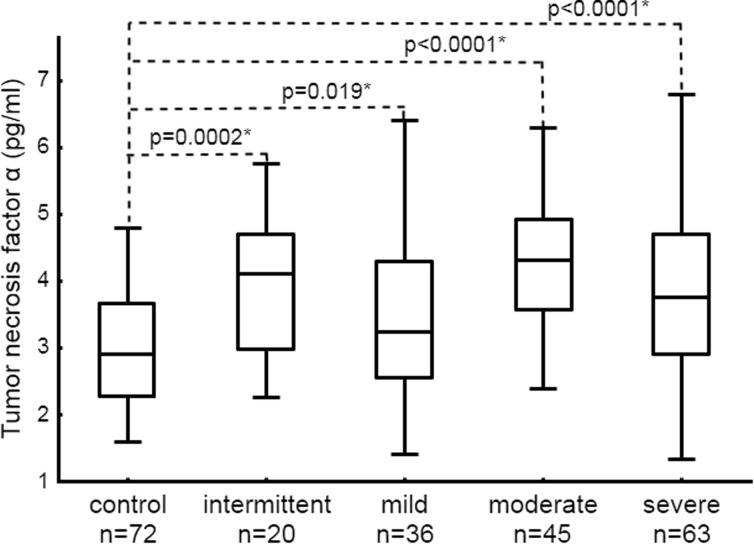
Distribution of tumor necrosis factor α in controls and patients with intermittent, persistent mild, moderate, and severe asthma. Data is presented as median, interquartile range, and maximum and minimum values. Numbers on the graph represent *p* values in comparison to control. Statistically significant differences are marked with an *asterisk*.

In asthma, we demonstrated 62% higher plasma levels of IL-6 and 35% higher TNFα, also after adjustment for potential confounders (*β* = 0.43 [95% CI, 0.37–0.49] and *β* = 0.37 [95% CI, 0.31–0.43], respectively). There were no differences between allergic and non-allergic subjects in the two cytokines (Table 1). Plasma IL-6 and TNFα were higher in persistent and sporadic asthmatics (Figs. 1 and 2). As shown in Table 2, asthmatics had a markedly higher risk of elevated IL-6 (OR 5.48 [95% CI, 4.7–7.0]) and TNFα (OR 3.93 [95% CI, 2.8–5.1]), compared with the controls (cut-offs, 4.08 and 3.66 pg/ml, respectively).

**Table 2:** Odds Ratios (ORs) with 95% Confidence Intervals (95% CI) for Asthmatics of Having Interleukin 6 and Tumor Necrosis Factor α Above 75th Percentile of Control Values with Subjects Below This Cutoff Point as a Reference Group, with Adjustment for Age, Sex, and Body Mass Index

	Interleukin 6 >4.08 pg/ml (75th percentile of the control values)		Tumor necrosis factor α >3.66 pg/ml (75th percentile of the control values)	
Control, numbers	18		18	
Asthma, numbers	106		93	
OR (95% CI)	5.48* (4.7–7.0)		3.93* (2.8–5.1)	
Allergic asthma, numbers	55		56	
OR (95% CI)	4.23* (3.1–5.4)		4.42* (3.2–5.6)	
Non-allergic asthma, numbers	51		37	
OR (95% CI)	8.05* (6.8–10.9)		3.36* (2.2–4.5)	
	<50 years	≥50 years	<50 years	≥50 years
Control, numbers	12	6	7	11
Asthma, numbers	39	67	35	58
OR (95% CI)	2.6* (1.4–3.77)	11.96 *(9.8–14.1)	4.26* (2.78–5.74)	3.56* (2.34–4.78)
	Men	Women	Men	Women
Control, numbers	4	14	4	14
Asthma, numbers	26	80	26	67
OR (95% CI)	5.69* (3.4–7.9)	5.44* (4.2–6.7)	5.69* (3.4–7.9)	3.48* (2.44–4.52)
	Body mass index <25	Body mass index ≥25	Body mass index <25	Body mass index ≥25
Control, numbers	9	9	8	10
Asthma, numbers	43	63	37	56
OR (95% CI)	5.16* (3.5–6.8)	5.73* (4.2–7.2)	4.18* (2.71–5.65)	3.64* (2.42–4.86)

* Statistically significant

A weak, but significant, inverse association was documented in asthmatics between IL-6 and the FEV_1_/VC ratio, also after adjustment for potential confounders (*β* = −0.13 [95% CI, −0.2 to −0.05]). IL-6 was associated in the asthma group with age (*β* = 0.19 [95% CI, 0.11–0.27]) and BMI (*β* = 0.25 [95% CI, 0.17–0.32]), as well as hsCRP (*β* = 0.6 [95% CI, 0.54–0.67]), fibrinogen (*β* = 0.29 [95% CI, 0.22–0.37]), TNFα (*β* = 0.29 [95% CI, 0.22–0.37]), and ETP (*β* = 0.35 [95% CI, 0.28–0.42]).

Among all parameters describing computational assessment of thrombin dynamics, IL-6 was associated only with thrombin-α_2_-macroglobulin complex formation (*β* = 0.19 [95% CI, 0.11–0.28]). On the other hand, none of the studied fibrinolytic parameters were related to this cytokine.

Co-morbidities did not influence IL-6 levels, but its higher values were observed in asthmatics receiving estrogens (*n* = 19, 5.24 [4.38–7.48] *vs.* 4.51[3.48–5.64] pg/ml, *p* = 0.04), beta adrenoreceptor antagonists (*n* = 21, 5.5 [4.43–6.98] *vs.* 4.51 [3.48–5.62] pg/ml, *p* = 0.01), and montelukast (*n* = 40, 5.16[4.05–6.24] *vs.* 4.5[3.46–5.48] pg/ml, *p* = 0.04). Higher IL-6 in asthma subjects on antileukotriene medication was also documented after adjustment for asthma severity and other potential confounders (*β* = 0.17 [95% CI, 0.09–0.25]).

IL-6 was associated with hsCRP in the control group (*β* = 0.43 [95% CI, 0.29–0.57]), TNFα (*β* = 0.43 [95% CI, 0.32–0.54]), and prothrombin activity (*β* = 0.31 [95% CI, 0.17–0.45]), as well as maximum prothrombin conversion rate (*β* = 0.27 [95% CI, 0.15–0.38] and thrombin-antithrombin complex formation (*β* = 0.29 [95% CI, 0.14–0.44]).

As expected, TNFα was associated with hsCRP in asthmatics (*β* = 0.33 [95% CI, 0.25–0.41]) and controls (*β* = 0.33 [95% CI, 0.18–0.48]). In asthma only, however, this cytokine was related to the prothrombin activity (*β* = 0.18 [95% CI, 0.1–0.26]), ETP (*β* = 0.15 [95% CI, 0.07–0.23]), and thrombin peak (*β* = 0.16 [95% CI, 0.08–0.23]). All these associations remained significant after correction for BMI, age, and sex in linear regression models. The magnitude of the impact of IL-6 on ETP, compared with that of TNFα, was larger (*p* = 0.03). Moreover, a multiple linear regression model showed that several laboratory parameters independently determined ETP in asthmatics, including hsCRP (*β* = 0.13 [95% CI, 0.03–0.23]) and IL-6 (*β* = 0.17 [95% CI, 0.08–0.26]) but not TNFα (Table 3).

**Table 3:** Multiple Linear Regression Models for Endogenous Thrombin Potential and Clot Lysis Time Taking into Account Interleukin 6, Tumor Necrosis Factor α, and Periostin

Endogenous thrombin potential						
	Control			Asthma		
	*β*	95% CI	*R* ^2^	*β*	95% CI	*R* ^2^
Age, years			0.4	0.03	−0.05 to 0.11	0.37
Body mass index, kg/m^2^	0.16^a^	0.01 to 0.31		0.2^a^	0.12 to 0.28	
FEV_1_/VC				–0.09^a^	−0.17 to −0.01	
Blood platelet count, 10^3^/μl				0.17^a^	0.09 to 0.25	
Total cholesterol, mmol/l				0.16^a^	0.08 to 0.24	
Factor II, %	0.38^a^	0.23 to 0.53		0.19^a^	0.12 to 0.26	
hsCRP, mg/l	0.15	−0.01 to 0.31		0.13^a^	0.03 to 0.23	
Immunoglobulin E (log10), IU/ml	0.26^a^	0.12 to 0.40				
Interleukin 6, pg/ml	0.01	−0.16 to 0.18		0.17^a^	0.08 to 0.26	
Tumor necrosis factor α, pg/ml	0.07	−0.08 to 0.22		0.04	−0.04 to 0.12	
Periostin, ng/ml				0.02	−0.06 to 0.1	
α_2_-macroglobulin, nmol/l	0.30^a^	0.14 to 0.45		0.34^a^	0.27 to 0.41	
Plasminogen, %				0.09^a^	0.02 to 0.16	
Platelet factor 4, ng/ml				0.01	−0.06 to 0.8	
Adjustment statistics	*F* = 3.29, *p* = 0.009			*F* = 6.9, *p* < 0.001		
Clot lysis time						
	Control			Asthma		
	*β*	95% CI	*R* ^2^	*β*	95% CI	*R* ^2^
FEV_1_/VC			0.51	−0.11^a^	−0.19 to −0,03	0.25
Blood platelet count, 10^3^/μl	0.14^a^	0.01 to 0.27				
White blood cells, 10^3^/μl	0.15^a^	0.03 to 0.27		0.01	−0.07 to 0.09	
Total cholesterol, mmol/l	0.13^a^	0.01 to 0.25		0.15^a^	0.07 to 0.23	
Triglycerides, mmol/l	0.19^a^	0.16 to 0.31				
Glucose, mmol/l	0.2^a^	0.06 to 0.34				
hsCRP, mg/l	−0.21^a^	−0.35 to −0.07		0.08	−0.02 to 0.18	
Immunoglobulin E (log10), IU/ml				−0.23^a^	−0.31 to −0.15	
Antithrombin, %				−0.11^a^	−0.19 to −0,03	
ETP, nmol/l thrombin x min.	−0.21^a^	−0.33 to −0.09		0.17^a^	0.12 to 0.26	
Plasminogen, %	0.2^a^	0.08 to 0.32		0.06	−0.02 to 0.14	
Interleukin 6, pg/ml	−0.002	−0.152 to 0.148		−0.05	−0.15 to 0.05	
Tumor necrosis factor α, pg/ml	0.31^a^	0.16 to 0.46		0.15^a^	0.07 to 0.23	
Periostin, ng/ml				0.04	−0.04 to 0.12	
Antiplasmin, %	0.3^a^	0.17 to 0.43				
Plasminogen activator inhibitor-1, ng/ml	0.35^a^	0.22 to 0.48		0.28^a^	0.2 to 0.36	
P-selectin, ng/ml	−0.26^a^	−0.4 to −0.12		−0.2^a^	−0.28 to −0.12	
Adjustment statistics	*F* = 4.2, *p* = 0.0006			*F* = 4.37, *p* < 0.001		

The resulting standardized regression coefficient (*β*) (with 95% confidence intervals [95% CI]) for a factor (independent variable) indicates the increase/decrease in standard deviations (SDs) of dependent variable, when that particular factor increases with 1 SD and all other variables in the model are unchanged

*FEV*_*1*_ */VC* forced expiratory volume in 1 s/vital capacity, *hsCRP* high-sensitivity C-reactive protein, *ETP* endogenous thrombin potential

^a^Statistically significant

Among parameters describing computational assessment of thrombin dynamics, TNFα was related in asthmatics only to the thrombin-α_2_-macroglobulin complex formation (*β* = 0.15 [95% CI, 0.06–0.24]), while in controls, it was associated with the total prothrombin converted and thrombin-antithrombin complex formation (*β* = 0.31 [95% CI, 0.16–0.46], both).

In both groups of subjects, there were no associations of TNFα with platelet activation markers, as well as CLT, PAI-1, and antiplasmin. A weak inverse relationship was documented for TNFα and plasminogen in asthma (*β* = −0.1 [95% CI, −0.18 to −0.03]) and in the controls (*β* = −0.26 [95% CI, −0.38 to −0.15]). However, as shown in Table 3, multiple linear regression models demonstrated that TNFα might be an independent predictor of longer CLT in asthma and control, although a direct association between both these variables was not documented in linear univariate regression models.

Medications used and co-morbidities analyzed separately had no impact on plasma TNFα. However, men with arterial hypertension (*n* = 16) had higher levels of TNFα than those without this co-morbidity (*n* = 26), (4.53 [3.57–5.05] *vs.* 3.8 [3.12–4.5] pg/ml, *p* = 0.03).

### Periostin

Serum concentration of periostin was measured only in asthmatics. Patients treated with oral corticosteroids (*n* = 34) had lower periostin levels than those not receiving this medication (0.35 [0.3–0.48] *vs.* 0.45 [0.33–0.73] ng/ml, *p* = 0.02). Other asthma medications and co-morbidities had no impact on periostin concentration. There was no difference among all asthma severity groups after the exclusion of subjects receiving oral corticosteroids. Periostin did not correlate with eosinophilia, blood IgE, or other basic laboratory tests. A positive association of periostin with α_2_-macroglobulin was observed also after correction for potential confounders (*β* = 0.16 [95% CI, 0.07–0.26]). There were inverse correlations of periostin with blood platelet count (*β* = −0.12 [95% CI, −0.21 to −0.04]) and total cholesterol (*β* = −0.27 [95% CI, −0.36 to −0.18]).

## DISCUSSION

The present study is the first to show that a hypercoagulable state in asthma, characterized by enhanced plasma thrombin formation, is associated with increased blood levels of inflammatory cytokines, IL-6 and TNFα. This indicates that the systemic low-grade inflammatory state contributes to the activation of blood coagulation in asthmatics, providing additional evidence for close links between inflammation and coagulation. We found no evidence for the involvement of periostin, a marker of Th2-mediated activation, in the prothrombotic alterations in asthma. The current study indicates that asthma, like other inflammatory diseases, *e.g.*, chronic obstructive pulmonary disease or inflammatory bowel disease [24, 25], is associated with prothrombotic changes in circulating blood that are largely driven by acute phase inflammatory mechanisms.

### Inflammatory Cytokines

We have shown in this study that IL-6 and TNFα were higher in asthmatics and associated with the total amount of thrombin generated using the well-established CAT assay. The effect of IL-6 on thrombin formation was stronger in asthmatics than that observed for TNFα. This indicates that IL-6-mediated inflammation is a major driver of increased hypercoagulability in asthma, similarly to several other diseases including atherosclerotic vascular disease [26–28]. It remains to be established, however, whether asthma also predisposes to this disease [29, 30].

The precise mechanisms underlying the associations between inflammatory cytokines and thrombin formation in asthma remain unclear. The most tempting hypothesis might be that systemic inflammation largely driven by IL-6 and reflected by elevated CRP stimulates the activation of procoagulant mechanisms, leading to the enhanced thrombin formation, as it was shown in subjects with acute coronary syndrome [13]. IL-6 *inter alia* induces fibrinogen synthesis by hepatocytes and other cells and is involved in iron dysregulation, in particular by its ability to induce hepcidin biosynthesis [31]. Procoagulant effects of IL-6 and TNFα involve also an increased expression of TF on monocytes, endothelial, dendritic, and vascular smooth muscle cells [32, 33]. TF is a surface receptor for FVIIa and plays a key role in the initiation of blood coagulation, leading to thrombin formation [34]. The TF signaling proceeds with the sequential generation of coagulant mediators (FVIIa, FXa, and FIIa: active serine proteases) and fibrin production, all of which are procoagulant but also proinflammatory [35]. A molecular link between coagulation and inflammation might be explained by specific G protein-coupled protease-activated receptors (PARs), designated PAR 1–4, which are expressed on variable airway cells, including smooth muscles and epithelial cells, as well as inflammatory infiltrating cells (for review, see [35]). Activation of these receptors by thrombin, FXa, and TF/FVIIa leads to the overproduction of, *e.g.*, IL-6, IL-8, platelet-derived growth factor, and P-selectin and also might participate in airway remodeling, another important feature of asthma, which develops independently of corticosteroid therapy and severity of bronchial inflammation [36, 37]. Notably, anticoagulant therapy is not used in asthma treatment nowadays, although interventions with anticoagulants such as fondaparinux (FXa inhibitor) and hirudin (thrombin inhibitor), as well as the use of tPA and uPA improved the disturbed pulmonary hemostatic balance and concurrently diminished airway inflammation and improved asthma parameters, including features of airway remodeling, in experimental settings [3]. Favorable effects of inhaled heparin on airway inflammation and asthma symptoms observed in humans might be attributable in part to the suppression of excessively enhanced thrombin formation and elimination of harmful thrombin-mediated cellular inflammatory effects [3].

In our previous study, we also employed a computational analysis of thrombin generation curves to investigate the balance of pro- and anticoagulant mechanisms during thrombin generation [8]. This equilibrium was extensively disturbed by increased prothrombin conversion and reduced thrombin inactivation in asthma patients. Surprisingly, both analyzed inflammatory cytokines in asthmatics were positively associated only with thrombin-α_2_-macroglobulin complex formation. This observation suggests that IL-6 and TNFα might increase the capacity to buffer active thrombin by its facilitated binding to the α_2_-macroglobulin, which was also higher in asthmatics than in the controls. This might suggest an unexpected anti-inflammatory mode of action observed for the two cytokines. Further studies are needed to validate this observation.

Inflammation might have also an impact on decreased clot susceptibility to lysis. Impaired fibrinolysis has been documented in variable inflammatory diseases [38], including asthma [8]. Clot formation is affected by a plethora of circulating plasma molecules, *e.g.*, IL-1, and its properties involving the pore size, fibrin diameter, rigidity, and ease of fibrinolysis, which may vary in inflammation [38]. We did not observe any association between both inflammatory cytokines and CLT in our study, which is a global plasma test of fibrinolysis successfully used in several disease states to document impaired lytic mechanisms in patients [39]. As we showed previously, impaired fibrinolysis in asthma was largely driven by increased PAI-1 [8]. In the present study, IL-6 and TNFα had no direct impact on plasma PAI-1 antigen concentrations. However, a negative link between TNFα and plasminogen suggests that this cytokine might also contribute, at least to some extent, to the impaired fibrinolysis. This concept has been supported by constructed multiple linear regression models, which demonstrated that TNFα in cooperation with other laboratory variables affected CLT in asthmatics but also (even stronger) in the controls. Our observation is in line with the result of *in vivo* experiments, in which the infusion of recombinant TNFα was capable of eliciting the release of PAI-1 into the circulation [18]. A mouse model showed another piece of evidence for a potential link between PAI-1and TNFα [40]. Inactivation of TNFα receptors results in significantly reduced plasma PAI-1 levels in this study.

### Periostin

Blood periostin levels in our study were not associated with asthma severity or eosinophilia. These findings are in accordance with the results of Wagener et al. [41], who found that the serum periostin was a weak predictor of eosinophilic asthma in mild-to-moderate asthma. A similar observation was published by Górska et al. [42]. This study showed no difference in this protein between asthma and control subjects, as well as no correlation with asthma inflammatory type. In our data, periostin was also not associated with any of the measured parameters of thrombin formation. A positive association of α_2_-macroglobulin with periostin is a new observation, which requires a comment. α_2_-macroglobulin functions as a universal protease inhibitor in blood and is capable of binding various host or foreign peptides and particles, cytokines, and growth factors. It serves as an unspecific defense barrier against pathogens in the plasma and tissues [43]. A strong association observed between periostin and α_2_-macroglobulin might indicate that the excess of this protein would be bound to the α_2_-macroglobulin.

### Study Limitations

The study group was relatively small. We determined each variable at a single time point and therefore we cannot exclude changes of the variables studied over time. Periostin was measured only in asthmatics, which made comparisons impossible between the studied groups. We did not analyze Th2 cytokines, such as IL-4, IL-5, or IL-13, and therefore we cannot exclude a certain involvement of Th2 response in the regulation of blood coagulation in asthma. The assessment of medication-induced alterations to inflammatory markers was beyond the scope of our study. Interestingly, we observed that treatment with systemic corticosteroids was associated with lower periostin levels, while those on montelukast had higher IL-6 in plasma, independently from the asthma severity and other potential confounders, including age, BMI, or co-morbidities. Surprisingly, an unfavorable effect of antileukotriene medication was also observed in relation to the ETP and CLT, as described in our previous publication [8]. We did not determine other potential modulators of coagulation and fibrinolysis, *e.g.*, genetic polymorphisms. Given prothrombotic abnormalities in first-degree relatives of patients with venous thrombosis [44], we cannot exclude that a prothrombotic state observed in asthma is to some extent genetically determined [45]. Statistical associations reported here may not necessarily indicate cause-effect relationships. Finally, clinical relevance of prothrombotic alterations in asthmatics and their relation to the inflammation in terms of thromboembolic risk as well as their molecular mechanisms remain to be established.

## CONCLUSION

Our study demonstrates that enhanced thrombin generation in asthma might be a reason of systemic inflammatory state and is, at least partially, driven by inflammatory cytokines, such as IL-6 and TNFα. On the other hand, impaired fibrinolysis is not directly associated with their levels. Further studies are needed to better characterize links between inflammation and prothrombotic alterations in asthmatics.

## References

[1] Van der Poll, T., E. de Jonge, and H. ten Cate. 2000–2013. Cytokines as regulators of coagulation. In: *Madame Curie Bioscience Database [Internet]*. Austin (TX): Landes Bioscience. Available from: http://www.ncbi.nlm.nih.gov/books/NBK6207/. Accessed 14 July 2016.

[2] Hartley R, Berair R, Brightling CE (2014). Severe asthma: Novel advances in the pathogenesis and therapy. Polskie Archiwum Medycyny Wewnętrznej.

[3] De Boer JD, Majoor CJ, van’t Veer C, Bel EH, van der Poll T (2012). Asthma and coagulation. Blood.

[4] Chung WS, Lin CL, Ho FM, Li RY, Sung FC, Kao CH, Yeh JJ (2014). Asthma increases pulmonary thromboembolism risk: A nationwide population cohort study. The European Respiratory Journal.

[5] Majoor CJ, Kamphuisen PW, Zwinderman AH, Ten Brinke A, Amelink M, Rijssenbeek-Nouwens L, Sterk PJ, Büller HR, Bel EH (2013). Risk of deep vein thrombosis and pulmonary embolism in asthma. The European Respiratory Journal.

[6] Chung WS, Lin CL, Chen YF, Ho FM, Hsu WH, Kao CH (2014). Increased stroke risk among adult asthmatic patients. European Journal of Clinical Investigation.

[7] Onufrak SJ, Abramson JL, Austin HD, Holguin F, McClellan WM, Vaccarino LV (2008). Relation of adult-onset asthma to coronary heart disease and stroke. The American Journal of Cardiology.

[8] Bazan-Socha S, Mastalerz L, Cybulska A, Zareba L, Kremers R, Zabczyk M, Pulka G, Iwaniec T, Hemker C, Undas A (2016). Asthma is associated with enhanced thrombin formation and impaired fibrinolysis. Clinical and Experimental Allergy.

[9] Sneeboer MM, Majoor CJ, de Kievit A, Meijers JC, van der Poll T, Kamphuisen PW, Bel EH (2016). Prothrombotic state in patients with severe and prednisolone-dependent asthma. The Journal of Allergy and Clinical Immunology.

[10] Borish L, Rosenwasser LJ, Adkinson NF, Bochner B, Busse W, Holgate S, Lemanske R, Simons FE (2009). Cytokines in allergic inflammation. Middleton’s Allergy, Principles and Practice.

[11] Yokoyama A, Kohno N, Fujino S, Hamada H, Inoue Y, Fujioka S, Ishida S, Hiwada K (1995). Circulating interleukin-6 levels in patients with bronchial asthma. American Journal of Respiratory and Critical Care Medicine.

[12] Stouthard JM, Levi M, Hack CE, Veenhof CH, Romijn HA, Sauerwein HP, van der Poll T (1996). Interleukin-6 stimulates coagulation, not fibrinolysis, in humans. Thrombosis and Haemostasis.

[13] Undas A, Szułdrzyński K, Brummel-Ziedins KE, Tracz W, Zmudka K, Mann KG (2009). Systemic blood coagulation activation in acute coronary syndromes. Blood.

[14] Van der Poll T, Levi M, van Deventer SJ, ten Cate H, Haagmans BL, Biemond BJ, Büller HR, Hack CE, ten Cate JW (1994). Differential effects of anti-tumor necrosis factor monoclonal antibodies on systemic inflammatory responses in experimental endotoxemia in chimpanzees. Blood.

[15] Silvestri M, Bontempelli M, Giacomelli M, Malerba M, Rossi GA, Di Stefano A, Rossi A, Ricciardolo FL (2006). High serum levels of tumour necrosis factor-alpha and interleukin-8 in severe asthma: Markers of systemic inflammation?. Clinical and Experimental Allergy.

[16] Van der Poll T, Büller HR, ten Cate H, Wortel CH, Bauer KA, van Deventer SJH, Hack CE, Sauerwein HP, Rosenberg RD, ten Cate JW (1990). Activation of coagulation after administration of tumor necrosis factor to normal subjects. The New England Journal of Medicine.

[17] Van der Poll T, Levi M, Büller HR, van Deventer SJH, de Boer JP, Hack CE, ten Cate JW (1991). Fibrinolytic response to tumor necrosis factor in healthy subjects. The Journal of Experimental Medicine.

[18] Van Hinsbergh VW, Bauer KA, Kooistra T, Kluft C, Dooijewaard G, Sherman ML, Nieuwenhuizen W (1990). Progress of fibrinolysis during tumor necrosis factor infusions in humans. Concomitant increase in tissue-type plasminogen activator, plasminogen activator inhibitor type-1, and fibrin(ogen) degradation products. Blood.

[19] Jia G, Erickson RW, Choy DF, Mosesova S, Wu LC, Solberg OD, Shikotra A, Carter R, Audusseau S, Hamid Q, Bradding P, Fahy JV, Woodruff PG, Harris JM, Arron JR, Bronchoscopic Exploratory Research Study of Biomarkers in Corticosteroid-Refractory Asthma (BOBCAT) Study Group (2012). Periostin is a systemic biomarker of eosinophilic airway inflammation in asthmatic patients. The Journal of Allergy and Clinical Immunology.

[20] Kanemitsu Y, Matsumoto H, Izuhara K, Tohda Y, Kita H, Horiguchi T, Kuwabara K, Tomii K, Otsuka K, Fujimura M, Ohkura N, Tomita K, Yokoyama A, Ohnishi H, Nakano Y, Oguma T, Hozawa S, Nagasaki T, Ito I, Oguma T, Inoue H, Tajiri T, Iwata T, Izuhara Y, Ono J, Ohta S, Tamari M, Hirota T, Yokoyama T, Niimi A, Mishima M (2013). Increased periostin associates with greater airflow limitation in patients receiving inhaled corticosteroids. The Journal of Allergy and Clinical Immunology.

[21] Del Prete G, de Carli M, Lammel RM, D’Elios MM, Daniel KC, Giusti B, Abbate R, Romagnani S (1995). Th1 and Th2 T-helper cells exert opposite effects on procoagulant activity and tissue factor production by human monocytes. Blood.

[22] Global Initiative for Asthma (GINA) Report. Revised 2016. *Global Strategy for Asthma Management and Prevention*. Available from: www.ginasthma.org. Accessed 14 July 2016.

[23] Hemker HC, Kremers R (2013). Data management in thrombin generation. Thrombosis Research.

[24] Jankowski M, Undas A, Kaczmarek P, Butenas S (2011). Activated factor XI and tissue factor in chronic obstructive pulmonary disease: Links with inflammation and thrombin generation. Thrombosis Research.

[25] Owczarek D, Cibor D, Sałapa K, Głowacki MK, Mach T, Undas A (2013). Reduced plasma fibrin clot permeability and susceptibility to lysis in patients with inflammatory bowel disease: A novel prothrombotic mechanism. Inflammatory Bowel Diseases.

[26] Yudkin JS, Kumari M, Humphries SE, Mohamed-Ali V (2000). Inflammation, obesity, stress and coronary heart disease: Is interleukin-6 the link?. Atherosclerosis.

[27] Jones KG, Brull DJ, Brown LC, Sian M, Greenhalgh RM, Humphries SE, Powell JT (2001). Interleukin-6 (IL-6) and the prognosis of abdominal aortic aneurysms. Circulation.

[28] Swerdlow DI, Holmes MV, Kuchenbaecker KB, Engmann JEL, Shah T, Sofat R, Guo YR, Chung C, Peasey A, Ster RP, Mooijaart SP, Ireland HA, Leusink M, Langenberg C, Li K, Palmen J, Howard P, Cooper JA, Drenos F, Hardy J, Nalls MA, Li YR, Lowe G, Stewart M, Bielinski SJ, Peto J, Timpson NJ, Gallacher J, Dunlop M, Houlston R, Tomlinson I, Tzoulaki I, Luan J, Boer JMA, Forouhi NG, Onland-Moret NC, van der Schouw YT, Schnabel RB, Hubacek JA, Kubinova R, Baceviciene M, Tamosiunas A, Pajak A, Topor-Madry R, Malyutina SA, Baldassarre D, Sennblad B, Tremoli E, de Faire U, Ferrucci L, Bandenelli S, Tanaka T, Meschia JF, Singleton A, Navis G, Leach IM, Bakker SJL, Gansevoort RT, Ford I, Epstein SE, Burnett MS, Devaney JM, Jukema JW, Westendorp RGJ, de Borst GJ, van der Graaf Y, de Jong PA, Maitland-van der Zee AH, Klungel OH, de Boer A, Doevendans PA, Stephens JW, Eaton CB, Robinson JG, Manson JE, Fowkes FGR, Frayling TM, Price JF, Whincup PH, Morris RW, Lawlor DA, Smith GD, Ben-Shlomo Y, Redline S, Lange LA, Kumari M, Wareham NJ, Verschuren WMM, Benjamin EJ, Whittaker JC, Hamsten A, Dudbridge F, Delaney JAC, Wong A, Kuh D, Hardy R, Castillo BA, Connolly JJ, van der Harst P, Brunner EJ, Marmot MG, Wassel CL, Humphries SE, Talmud PJ, Kivimaki M, Asselbergs FW, Voevoda M, Bobak M, Pikhart H, Wilson JG, Hakonarson H, Reiner AP, Keating BJ, Sattar N, Hingorani AD, Casas JP (2012). The interleukin-6 receptor as a target for prevention of coronary heart disease: A Mendelian randomisation analysis. Lancet.

[29] Onufrak S, Abramson J, Vaccarino V (2007). Adult-onset asthma is associated with increased carotid atherosclerosis among women in the Atherosclerosis Risk in Communities (ARIC) study. Atherosclerosis.

[30] Iribarren C, Tolstykh IV, Eisner MD (2004). Are patients with asthma at increased risk of coronary heart disease?. International Journal of Epidemiology.

[31] Duan HO, Simpson-Haidaris PJ (2003). Functional analysis of interleukin 6 response elements (IL-6REs) on the human gamma-fibrinogen promoter: Binding of hepatic Stat3 correlates negatively with transactivation potential of type II IL-6REs. The Journal of Biological Chemistry.

[32] Grignani G, Maiolo A (2000). Cytokines and hemostasis. Haematologica.

[33] Scarpati EM, Sadler JE (1989). Regulation of endothelial cell coagulant properties. Modulation of tissue factor, plasminogen activator inhibitors, and thrombomodulin by phorbol 12-myristate 13-acetate and tumor necrosis factor. The Journal of Biological Chemistry.

[34] Furie B, Furie BC (2008). Mechanisms of thrombus formation. NEJM.

[35] Chu AJ (2005). Tissue factor mediates inflammation. Archives of Biochemistry and Biophysics.

[36] Shimizu S, Gabazza EC, Hayashi T, Ido M, Adachi Y, Suzuki K (2000). Thrombin stimulates the expression of PDGF in lung epithelial cells. American Journal of Physiology. Lung Cellular and Molecular Physiology.

[37] Moffatt JD, Page CP, Laurent GJ (2004). Shooting for PARs in lung diseases. Current Opinion in Pharmacology.

[38] Kell DB, Pretorius E (2015). The simultaneous occurrence of both hypercoagulability and hypofibrinolysis in blood and serum during systemic inflammation, and the roles of iron and fibrin(ogen). Integrative Biology.

[39] Pankiw-Bembenek O, Zalewski J, Goralczyk T, Undas A (2012). A history of early stent thrombosis is associated with prolonged clot lysis time. Thrombosis and Haemostasis.

[40] Samad F, Uysal T, Wiesbrock S, Pandey M, Hotamisligil S, Loskutoff J (1999). Tumor necrosis factor α is a key component in the obesity-linked elevation of plasminogen activator inhibitor 1. Proceedings of the National Academy of Sciences of the United States of America.

[41] Wagener AH, de Nijs SB, Lutter R, Sousa AR, Weersink EJ, Bel EH, Sterk PJ (2015). External validation of blood eosinophils, FE(NO) and serum periostin as surrogates for sputum eosinophils in asthma. Thorax.

[42] Górska K, Maskey-Warzęchowska M, Nejman-Gryz P, Korczyński P, Prochorec-Sobieszek M, Krenke R (2016). Comparative study of periostin expression in different respiratory samples in patients with asthma and chronic obstructive pulmonary disease. Polskie Archiwum Medycyny Wewnętrznej.

[43] Borth W (1992). Alpha 2-macroglobulin, a multifunctional binding protein with targeting characteristics. The FASEB Journal.

[44] Kyrle PA (2014). Venous thrombosis: Who should be screened for thrombophilia in 2014?. Polskie Archiwum Medycyny Wewnętrznej.

[45] Undas A, Zawilska K, Ciesla-Dul M, Lehmann-Kopydłowska A, Skubiszak A, Ciepłuch K, Tracz W (2009). Altered fibrin clot structure/function in patients with idiopathic venous thromboembolism and in their relatives. Blood.

